# Current News and Opinion

**Published:** 1886-09

**Authors:** 


### Current News and Opinion.

### UNIVERSITIES OF EUROPE.

Of German Universities located outside of the German Empire, that of Prague (founded 1348), is the oldest, and that of Vienna (1365), follows next. Of those founded in Germany in the 14th century these still exist:

Heidelberg, founded............1386.

15th Century.

| | |
|---|---|
| Leipzig, founded.................1409. | Frieburg, founded..............1457. |
| Rostock, founded.................1419. | Tubingen, founded..............1477. |
| Greifswald, founded..............1456. | |

16th Century.

| | |
|---|---|
| Marbourg, founded............ 1527. | Olmutz, founded............... 1581. |
| Konigsburg, founded............1544. | Wurzburg, founded..............1582. |
| Jena, founded..................1558. | Graz, founded..................1586. |

17th Century.

| | |
|---|---|
| Giessen, founded...............1607. | Innsbruck, founded.............1672. |
| Kiel, founded .................1665. | Halle, founded.................1694. |

18th Century.

| | |
|---|---|
| Breslau, founded...............1702. | Erlangen, founded .............1743. |
| Gottingen, founded.............1737. | |

19th Century.

| | |
|---|---|
| Berlin, founded................1809. | Munchen, founded...............1826. |
| Bonn, founded..................1818. | |

The following named have been discontinued : Cologne (founded 1388), Erfurt (1392), Mainz (1477), Dillingen (1549), Helmstadt (1576), Altorf (1578), Paderborn (1615), Rinteln (1621), Osnabruck (1630), Linz (1636), Bamberg (1648), Herborn (1654), and Duisburg (1655). The University founded at Ingolstadt in 1472 was in 1802 removed to Landshut, and in 1826 to Munchen; that founded at Wittenberg in 1582 was removed to Halle in 1817, and that founded at Frankfurt in 1506, was removed to Breslau in 1811.

The number of students in attendance at the 20 Universities of Germany during the last scholastic year was 28,021, distributed as follows :

| | |
|---|---|
| Berlin.......................4,434. | Heidelberg...................1,036. |
| Leipzig......................3,060. | Greifswald...................1,016. |
| Munchen......................3,035. | Marbourg..................... 939. |
| Halle........................1,518. | Erlangen .................... 909. |
| Breslau......................1,425. | Konigsberg .................. 871. |
| Tubingen ....................1.403. | Strassburg................... 846. |
| Wurzburg ....................1,369. | Jena ........................ 655. |
| Frieburg.....................1,319. | Kiel......................... 542. |
| Bonn.........................1,293. | Giessen ..................... 513. |
| Gottingen ...................1,076. | Rostock...................... 313. |

Compared with the year 1880, the increase (33.4 %) of students amounts to 7,033.

### ARTIFICIAL SOCKETS.

The following humorous account of an operation by Dr. Morrison is extracted from a private letter to the editor, but it is so apropos to the article by Dr. Southworth, in this number, and it so clearly describes a case with which a number of western dentists are more or less familiar, and which we think antedates the cases of Dr. Younger, that the temptation to print it is irresistible. If the author of the letter is offended by its public use, the readers of this paragraph will please apologize to Dr. Geo. L. Field, of Detroit:

"A Right Superior Cuspid, instead of erupting in its proper place in the jaw, got on a tear, and when it undertook to fall into line found that the ranks were closed up. The "three months' man," as we used to say some few years ago, during the "unpleasantness," had served his time and had been mustered out of the service, and the fellow who was to take his place, and had "enlisted for the war" was, as I have said, off on a big drunk, or something of the kind. The order to "right dress" had been given, and had been obeyed. The gap was filled up, and Mr. R. Cuspid was left out in the cold. So he went nosing around for a place in the ranks, and in his blind hurry he ran *completely through* Mr. R. Central, just below the belt, in direct violation of the Queensbury rules. It made Mr. R. Central sick unto death. He tottered on his feet, and could hardly keep his place in the ranks. Drs. Spalding & Park, surgeons of the Odontological Regiment, attended upon the poor fellow, and gave it as their opinion that if Mr. Canine was only removed and sent to the guard house, or somewhere else, the chances for Mr. Incisor's recovery were, in their opinion, fairly good. Dr. Spalding and I had some correspondence about the matter, and I did not agree as to what would be the result. I gave it as my opinion that when Mr. Canine was made to vacate the space he was occupying in Mr. Incisor's body, Mr. I. would keel over and sleep with his fathers, and under the circumstances, that would be the best thing for him to do, as his place could be filled with a *dummy*, who would do for show, backed up with gold, like many another fellow now holding place in the regular army. The little lady who raised the company would not, however, listen to the advice of either party, and continued her search for some other surgeon, hoping for better results. In time she hit upon a man by the name of Morrison, of St. Louis, who took a different view of the case from any of us. He was a reckless kind of a chap, and took big chances. He suggested clearing the ranks of both the disabled man and the drunken brute who had run him through, and transferring from some other company a good man and true, one who had never been sick or wounded, and who would require no gold to back him up and make him stand well before the world. There was another company in the same oral cavity, only it stood lower than the one spoken of. These men were called *inferior*, but really they were quite as good as those who were their superiors, although they were somewhat demoralized, and the ranks out of line. There was one fellow so far out that there was no getting him back, and he was the cause of trouble to the rest of the men. So surgeon Morrison took charge of both companies and removed both men from the upper company, and with an implement of war called a bur bored a deep hole in the parade ground, which is sometimes called the alveolus. He then took the unruly member from this lower company by the scuff of the neck, and stood him in the place prepared for him above. The recruit took kindly to his new quarters, though he was for a time firmly bound to a couple of his comrades on each side of him. The thongs have now been long removed, and no one would ever suspect by looking at him that he ever belonged to any other company than the one in which he is now enlisted for the war. The young lady who commands these two companies is a rather frail, delicate girl, extremely sensitive, but is justly proud of what has been done for her.

"I got permission from her to let Prof. Taft make an examination of the case, and he apparently was as much mystified over the result as I was. There was absolutely *no periosteum* left where the old member stood. I forgot to say that the lower tooth which was removed to the upper jaw was a *cuspid.* Dr. M. ground it up and filled one side of it so as to make it more the shape of a central incisor, and he was quite successful in his efforts. The tooth to-day is apparently as solid as any tooth in the head, and does good service."

---

### BAKER'S CARIES.

BY PROF. DR. HESSE, LEIPZIG.

In the Dental Institute of this city I have the opportunity of seeing a great number of patients of the commercial and laboring classes, and nothing has caused me greater surprise than the deplorable condition of the teeth of bakers. They are attacked by dental caries to such a degree that I have been able, after some experience, to determine the profession of the patient in many cases by the condition of his teeth. The caries is soft and rapidly progressive. The principal parts attacked are the labial and buccal surfaces of the teeth, and caries spreads over the surface as well as it penetrates deep into the tissues. It commences at the cervical part, and progresses more rapidly toward the grinding surface than under the gums. The approximal surfaces do not seem to be attacked more than in other patients, but in most cases a great number of teeth were already so far gone that saving them could not be thought of, neither could one form a correct opinion as to the starting point of decay. All the patients I have examined are from seventeen to twenty-three years old, that is to say, of an age when ordinarily the teeth are still in good condition. We cannot doubt but that we have here to do with a disease that is in etiological connection with this calling, and the theory of decay which Miller has of late propounded offers a very satisfactory explanation of this fact. Miller has united the chemical and the parasitic theories, having proved that by "Spaltpilze" an acid is generated by which the calcareous salts of the enamel and dentine are dissolved. And he has furthermore established the important fact that the production of this acid takes place in the presence of fermentable carbo-hydrates. This last fact enables us to understand why bakers are so befallen with dental caries. As long as they work they inhale flour-dust, and I believe one who rises at two in the morning and goes to rest after a night of heavy physical labor, would hardly take sufficient care to keep his teeth clean, even were he one of a more elevated class of people. It is even questionable if the bakers would derive much benefit from the cleaning of their teeth, as the time of work is sufficiently long to enable the flour-dust to do its work of destruction.

I have only seen a few children of confectioners that might be compared to the bakers in this respect, although the degree of decay in their teeth was not so intense.

But it is to be expected that the millers may compete with the bakers, and it would be desirable to receive a communication in regard to this point.

—*Deutsche Monatschrift.*

### LEGISLATIVE QUACKERY.

In 1806, only eighty years ago, the Legislature of the State of New York passed a bill, and Governor Morgan Lewis signed and approved it, to pay to a notorious Hessian quack, $1,000 for his secret remedy for hydrophobia. The remedy was to be published and its efficacy assured before the money was paid, but the quack's name is found signed in receipt of the sum on the back of the comptroller's warrant. It should be remembered that the ordinary dose of calomel is ten grains, and of opium one-half a grain.

### CURE FOR THE BITE OF A MAD DOG.

1. Take one ounce of the jaw-bone of a dog, burned and pulverized or pounded to fine dust.

2. Take the false tongue of a newly-foaled colt; let that be also dried and pulverized ; and

3. Take one scruple of the verdigrease which is raised on the surface of old copper by laying in moist earth; the coppers of George I. or II. are the purest and best. Mix these ingredients together, and if the patient be an adult or full grown, take one common teaspoonful a day, and so in proportion for a child, according to its age. In one hour after take the filings of one-half of a copper of the above kind, if to be had; if not, then a small increased quantity of any baser metal of the kind, this to be taken in a small quantity of water. The next morning fasting, or before eating, repeat the same as before. This, if complied with after the biting of the dog, and before symptoms of madness, will effectually prevent any appearance of the disorder, but if after the symptoms shall appear, a physician must immediately be applied to to administer the following, viz.: Three drachms of the verdigrease of the kind before mentioned, mixed with half an ounce of calomel, to be taken at one dose. This quantity the physician need not fear to administer, as the reaction of the venom then diffused through the whole system of the patient neutralizes considerably the powerful quantity of the medicine; and secondly, if in four hours thereafter the patient is not completely relieved, administer four grains of pure opium, or 120 drops of liquid laudanum.

N. B.—The patient must be careful to avoid the use of milk for several days after taking any of the foregoing medicines. JOHN M. CROUSE.

---

### DENTAL JOURNALS WANTED.

The editor of this journal will pay cash for the following numbers of dental journals, or an exchange will be made with those who desire to complete their own files :

THE DENTAL REGISTER.

Vol. III, Nos. 1, 2, 3.

" VI, " 1.

AMERICAN JOURNAL OF DENTAL SCIENCE. (Third Series.)

Vol. II, No. 8.
" V, " 3, 7, 11.
" VI, " 2, 3, 5, 7, 10.
" VII, " 2, 3, 7.
" VIII, " 6, 7, 10.

### AMERICAN DENTAL ASSOCIATION.

The following is the list of officers of the Association elected at Niagara to serve for the coming year:

*President*—W. W. Allport, Chicago.

*First Vice-President*—Geo. W. McElhany, Columbus, Ga.

*Second Vice-President*—S. W. Dennis, San Francisco.

*Recording Secretary*—Geo. H. Cushing, Chicago.

*Corresponding Secretary*—A. W. Harlan, Chicago.

*Treasurer*—Geo. W. Keely, Oxford, Ohio.

*Executive Committee*—W. C. Wardlaw, Augusta, Ga., S. G. Perry, New York City, S. H. Guilford, Philadelphia.

Dr. E. T. Darby, Philadelphia, in place of J. N. Crouse, resigned.

Dr. A. W. Harlan, Chicago, in place of Dr. C. N. Pierce, resigned.

The Southern Dental Association was by vote invited to hold its meeting with the American Dental Association at Asheville, North Carolina, next year.

---

### AT NIAGARA.

Some of the new things exhibited at the Niagara meeting mark a decided advance in mechanics. Among them were the continuous-gum gas furnace of Dr. C. H. Land of Detroit. With this it is quite practicable for any competent dentist to make a better class of work than has been attempted by the average practitioner, both in plates and crowns.

Dr. G. W. Melotte's system of crown work attracted universal attention. The method of duplicating crown surfaces, by means of his impression compound, was strongly approved.

The crown and bridge work of Dr. Knapp, of New Orleans, was without doubt the most beautiful and artistic that has been exhibited. His nitrous-oxide compound blowpipe was a revelation to most of those who saw its possibilities.

Dr. S. G. Perry presented some improvements in the dental engine, which instantly caught the attention of every practical man.

---

"DANIEL'S MEDICAL JOURNAL for July says: In dismissing these (Pancoast, Shoemaker, and Atkinson) Southern and Western sympathizers, the Jefferson Medical College has lost its last hold on the patronage of the South and West."

We will back the new concern (the Medico-Chirurgical College) to the extent of our humble ability, and will urge the profession of the South and West to stand by them, as they stood by us in the affairs of the Congress, and suffered for opinion's sake.—*Weekly Medical Review.*

*Is* it the fact, then, that the Congress is a peculiarly Western and Southern affair? Our two exchanges seem to speak as if it were, and that is what is charged in the east. Why should sectionalism pervade even professional and scientific circles, and how is it that this feeling seems mainly confined to the South and West?

MRS. W., forty-five years of age, housewife, fell down stairs, striking her face against the railing, from which she sustained quite a severe contusion of the right cheek, with effusion of blood under the conjunctiva, from the outer canthus to the margin of the cornea. When first seen she was suffering from a slight frontal headache, for the relief of which the writer applied to the forehead not more than six or eight drops of sulphuric ether with a camel's hair brush. The patient at once said, "That is ether, and it is putting me to sleep," and in less than half a minute she was fully under the influence of the anæsthetic, remaining unconscious for several minutes, and upon recovery, and for some time afterwards, presented all the symptoms following profound and prolonged etherization. She could not have inhaled more than three to five drops of the ether, as it was immediately washed from her forehead.—*Med. Record.*

---

DR. WIDMARK, a Swedish surgeon, having as a patient a young girl in whom he was unable to detect the slightest pathological changes in the right eye, but who was completely blind on the other side, observing considerable defects in her teeth, sent her to M. Skogsborg, a dental surgeon, who found that all the upper and lower molars were completely decayed, and that in many of them the roots were inflamed. He extracted the remains of the molars on the right side, and in four days' time the sight of the right eye began to return, and on the eleventh day after the extraction of the teeth it had become quite normal. The diseased fangs on the other side were subsequently removed, lest they should cause a return of the ophthalmic affection.—*The Annals of Hygiene.*

---

AMONG THE hard worked pupils of the Paris public schools, the teeth become deteriorated in a few weeks after entering. The second dentition is often premature. These observations confirm the statements of Dr. J. L. Williams, who has given great attention to this subject. He has shown that any mental strain shows itself upon the teeth in a short time, in increased decay as well as in increased sensitiveness of the dentine. Dr. D. M. Parker has reported that the same changes are always apparent in those who are training for athletic trials. As there is not the slightest doubt of the accuracy of these observations, they show that these are matters which demand serious consideration from educators.—*Boston Medical and Surgical Journal.*

---

A CORRESPONDENT OF THE *Medical Brief* states that Mr. Savory, President of the Royal College of Surgeons, was recently going round his wards at St Bartholomew's Hospital, when he came to a case of hernia just operated upon. Turning to one of the students following him, he asked: "You have passed, have you not?" An affirmative answer was returned. "What is a good thing after an operation for hernia?" "A dose of opium." "How much would you give?" "Ten grains." "How would you give it?" "The liquid extract" "How much of it would you require to give you ten grains of opium?" "Half a dram." "It will not be long before they require an extra coroner in the district where you settle down," was the comment.

VOGEL relates the following in his "Diseases of Children;" I once treated an American lady, who still suckled her son, who was *two and a half years old*, till one morning, when the strongly-developed robust child was called to be nursed, he very kindly replied, "I thank you, mamma; the nursing is too tedious for me."—*Exchange.*

Well, it is safe to wager that the boy had never known what indigestion or diarrhœa was, and that he had sound teeth, and was likely to have more of the same kind.

---

DR. E. A. BOGUE, one of the secretaries of the Dental and Oral Section of the International Medical Congress, requests us to say that his address from August 10 to Jan. 1, 1887, will be 39 Boulevard Haussmann, Paris, France. After that date he will be at home at 29 East 2[illegible]th St , New York City.

It has been suggested that those who are willing to contribute papers upon any subject in which they may be specially interested shall inform either of the secretaries of such willingness, to be followed as soon as possible by an abstract of the paper.

---

DR. FRANK H. HAMILTON, the eminent surgeon, died in New York City on the 11th ult. For twenty years Buffalo was the home of Dr. Hamilton, and more than forty years ago, in connection with Austin Flint and James P. White, he founded the medical department of the University of Buffalo, and was its first Professor of Surgery. All three of these eminent men passed away within a brief period.

---

A YOUNG MAN entered the dispensary of the Chicago Polyclinic recently, and going up to the clerk held out one of the dispensary circulars, with the question: "Say! isn't this the hour for the diseases of women?" The clerk answered in the affirmative, when the young man said: "Well! I've got a disease of a woman and want to be treated."—*Jour. Am. Med. Association.*

---

THE LEGISLATURE of the State of Maryland has repealed the provision of the law which subjected graduates of other dental colleges to an examination by her own faculties. Such an act should never have been passed, as it gave rise to charges, no doubt false in themselves, but which had the semblance of probability.

---

MANY DENTISTS are exceedingly troubled by Hyperidrosis or excessive sweating. When this takes place upon the hands it makes their touch very disagreeable to many people who may be their patients. Washing the hands with a saturated solution of boracic acid is often effective as a cure.

---

"MY DEAR," said a frightened husband in the middle of the night, shaking his wife, "where did you put that bottle of strychnine?" "On the shelf next to the peppermint." "Oh, Lord!" he groaned, "I've swallowed it." "Well, for goodness sake," whispered his wife, "keep quiet or you'll wake the baby."

CHICAGO got its full share of the offices at the late election of the A. D. A. The President, the Recording Secretary, the Corresponding Secretary and one member of the Executive Committee hail from that city. The last two offices are worthily filled by Dr. Harlan.

---

"I AM AS much opposed to intemperance as anybody," said Smith, "but, nevertheless, liquor rightly used is a blessing to humanity. When I was ill last year, I really believe it saved my life." "Very likely," said Brown, "but how does that prove that liquor is a blessing to humanity ?"

---

THE TITLE OF DOCTOR was invented in the twelfth century, at the first establishment of the Universities. William Gordeina was the first person upon whom the title of Doctor of Medicine was bestowed. He received it from the college at Asti, in 1329.

---

AN EXCHANGE SAYS, "If a child does not thrive on fresh milk, boil it." This seems like very imprudent advice. In many localities there is an extreme prejudice against boiling babies, and a boiled baby is no better than a raw one, anyway.

---

"ARE YOU having much practice now ?" asked an old practitioner of a young graduate. "Yes, sir, a very absorbing one," was the answer. "Ah ! I am glad to hear of it. In what line is your practice, mostly ?" "Well, sir, particularly in economy."

---

CONVULSIONS may frequently be cut short like magic by turning the patient on his left side. The nausea as an after effect of chloroform or ether narcosis may generally be controlled in the same manner.

---

"DO YOU understand the language of flowers and of colors, doctor ?" said a lady to a bachelor physician. "This is a dandelion, and yellow means jealousy." "No, madam," was the answer, "yellow means biliousness."

---

MOLIERE said that doctors are those who pour out medicine about which they know little into bodies of which they know less, in order to cure diseases of which they know nothing at all.

---

THE MUCH-ADVERTISED "Cuticura Ointment" has been found to consist of petroleum jelly, colored green, perfumed with oil of bergamot, and containing two per cent. of carbolic acid.

---

DR. T. W. EVANS, of Paris, has received the decoration of the "Order of the Polar Star" from the King of Sweden, in acknowledgment of successful treatment at his hands.

---

DR. A. A. BLOUNT, formerly of Springfield, Ohio, who some years since went to Geneva, Switzerland, to practice, has returned to this country and will probably remain.

---

THE ODONTOGRAPHIC JOURNAL is growing larger, better and prettier with each number. It is beautifully printed, and its editing leaves little to be desired.

